# Hexagonal 2H-MoSe_2_ broad spectrum active photocatalyst for Cr(VI) reduction

**DOI:** 10.1038/srep35304

**Published:** 2016-10-13

**Authors:** Haipeng Chu, Xinjuan Liu, Baibai Liu, Guang Zhu, Wenyan Lei, Huigang Du, Junying Liu, Jianwei Li, Can Li, Changqing Sun

**Affiliations:** 1 Institute of Coordination Bond Metrology and Engineering, College of Materials Science and Engineering, China Jiliang University, Hangzhou 310018, China; 2 Anhui Key Laboratory of Spin Electron and Nanomaterials, Suzhou University, Suzhou 234000, China; 3 Research Center for Combustion and Environment Technology, Shanghai Jiao Tong University, Shanghai 200240, China; 4 Jiangsu Key Laboratory of Advanced Laser Materials and Devices, Hydrogen energy laboratory, Laboratory for Quantum Design of Functional Material, School of Physics and Electronic Engineering, Jiangsu Normal University, Xuzhou 221116, China

## Abstract

To make full use of the solar energy, exploring broad spectrum active photocatalysts has become one of the core issues for photocatalysis. Here we report a novel hexagonal 2H-MoSe_2_ photocatalyst with ultraviolet (UV)-visible-near infrared (NIR) light response for the first time. The results indicate that the MoSe_2_ displays excellent photo-absorption and photocatalytic activity in the reduction of Cr(VI) under UV and visible even NIR light irradiation. MoSe_2_ synthesized at pH value of 2 achieves the highest Cr(VI) reduction rates of 99%, 91% and 100% under UV, visible and NIR light irradiation, respectively, which should be attributed to its comparatively higher light absorption, efficient charge separation and transfer as well as relatively large number of surface active sites. The excellent broad spectrum active photocatalytic activity makes the MoSe_2_ to be a promising photocatalyst for the effective utilization of solar energy.

Agrowing number of contaminations such as heavy metal ions and organic chemical compounds in natural water have become a serious threat to environment and human health. Hexavalent chromium (Cr(VI)) is a most common contaminant, which is discharged from industries such as electroplating, leather tanning, metal finishing, textile manufacturing, steel fabricating, paint and pigments, fertilizing, and so on. It is highly toxic to most organisms when its concentration is above 0.05 mg l^−1^, and can cause the lung-cancer, chromeulcer, perforation of nasal septum and kidney-damage. Various techniques, such as adsorption, biosorption, electrocoagulation, ion exchange, membrane filtration, have been reported to remove the Cr(VI) from wastewater^1^,^2^. However, these techniques have some disadvantages, such as membrane fouling, high power consumption and cost for operation and maintenance.

The reduction of Cr(VI) to Cr(III) is considered as an efficient route to remove Cr(VI), because Cr(III) is less toxic and can be readily precipitated in aqueous solution in the form of Cr(OH)_3 _3. Semiconductor photocatalysis as a novel, economical and environmentally-friendly technique for the reduction of Cr(VI) has attracted considerable attention in recent years^4^,^5^,^6^,^7^. One of the major factors in photocatalysis is the limited light absorption of photocatalysts in the incident solar spectrum. Up to now, most of the photocatalysts such as TiO_2_, CuO, CdS, SnS_2_, AgCl:Ag, WO_3_ and metal-free g-C_3_N_4_ *et al*. are only active under ultraviolet (UV) or visible light irradiation^8^,^9^,^10^,^11^,^12^,^13^,^14^,^15^,^16^,^17^,^18^,^19^,^20^,^21^,^22^. UV and visible light make up only about 4% and 43% of the solar energy reaching the surface of the earth, respectively, while near-infrared (NIR) light constitutes more than 50%^23^,^24^,^25^. Nevertheless, up to now few efforts have been made to effectively utilize the NIR light^26^,^27^,^28^. Therefore, exploration of novel broad spectrum (UV, visible and NIR) responsive semiconductors with efficient and stable photocatalytic activity remains a challenge.

Transition metal dichalcogenides (TMDs) have attracted great interests due to their intriguing properties and potential applications in hydrogen evolution^29^, lithium/sodium batteries^30^, and photocatalysis^31^,^32^,^33^,^34^. Among these TMDs, MoSe_2_ with a narrow band gap of ~1.4 eV can harvest the solar energy in a very broad spectral region and has been employed as an efficient photocatalyst under UV, visible and NIR light irradiation^35^. Theoretical studies indicated that single layer MoSe_2_ is an ideal candidate for photocatalytic splitting of water to generate hydrogen in solar light irradiation^36^. So far, previous studies focused mainly on the visible light photocatalytic activity of MoSe_2_ in the degradation of dye. Unfortunately, there have been no reports on the photocatalytic activity of MoSe_2_ under UV and NIR light irradiation, especially for photocatalytic reduction of Cr(VI).

In this work, novel broad spectrum responsive MoSe_2_ was synthesized via a facile solvothermal method for the first time. The MoSe_2_ exhibits excellent photo-absorption in the whole light region and shows good photocatalytic activity in the reduction of Cr(VI) under UV, visible and NIR light irradiation. The photocatalytic mechanism was also studied in terms of a series of characterization and controlled experiments using hole scavengers.

## Results and Discussion

### Characterizations of MoSe_2_

**Figure 1:**
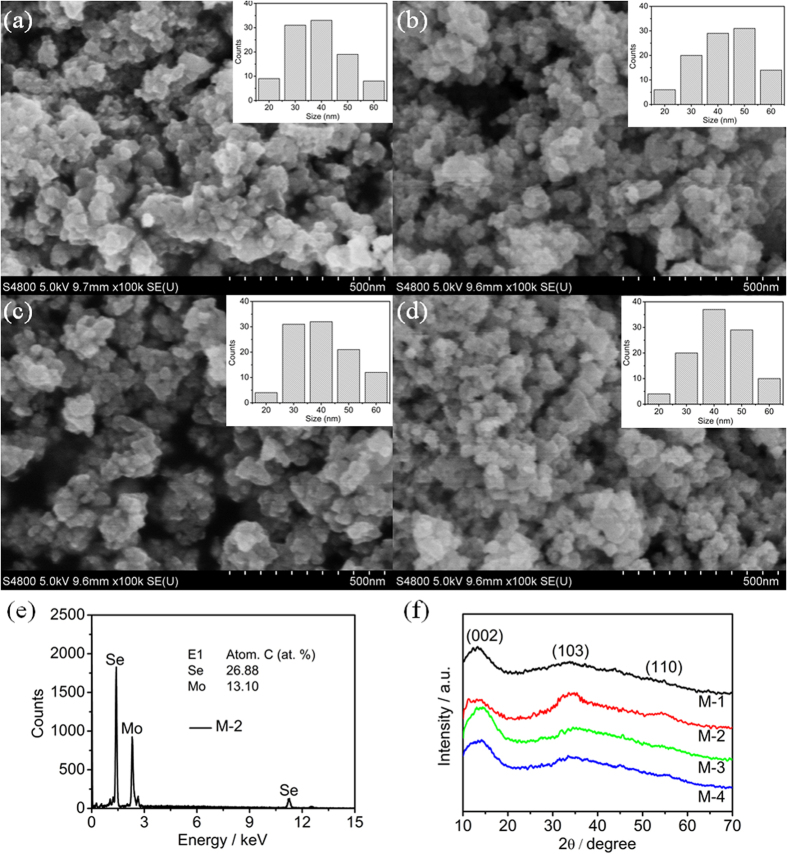
FESEM images of (**a**) M-1, (**b**) M-2, (**c**) M-3 and (**d**) M-4; (**e**) EDS spectrum and (**f**) XRD pattern of M-2. Scale bars: (**a–d**) 500 nm. Inset is the particle size distribution of (**a**) M-1, (**b**) M-2, (**c**) M-3 and (**d**) M-4.

Figure 1 shows the field-emission scanning electron microscopy (FESEM) images of M-1, M-2, M-3 and M-4. It is clearly observed that all the MoSe_2_ samples display the nanoparticle structures with a size range between 30 and 50 nm (inset of Fig. 1a–d), which indicates that the morphology of MoSe_2_ does not change when the pH value of the precursor solution increases from 1 to 4. However, the MoSe_2_ nanoparticles are aggregated when the pH value increases to 3 and 4. The M-2 was identified by energy dispersive X-ray spectroscopy (EDS) linked to FESEM, as shown in Fig. 1e. The atom ratio of Mo and Se is about 1:2, further indicating the formation of MoSe_2_. Figure 1f shows the X-ray diffraction (XRD) patterns of M-1, M-2, M-3 and M-4. The diffraction patterns of as-prepared MoSe_2_ samples show that all the peaks can be indexed to (002), (102) and (110) crystal planes of the hexagonal 2H-MoSe_2_ phase with space group *P*6_3_/*mmc* (JCPDS: 29–0914)^37^. No other impurity peaks for as-prepared MoSe_2_ samples are observed, which confirms that the as-prepared products are pure MoSe_2_. The XRD pattern of commercial MoSe_2_ (labeled as MoSe_2_ bulk) was also measured for comparison, as shown in Figure S1. It can be observed that the XRD patterns of as-prepared MoSe_2_ samples show relatively broader diffraction peaks compared with MoSe_2_ bulk, indicating that the as-prepared products are somewhat amorphous and have short range structural order^38^. This is in good agreement with the reported results^39^. The crystallinity does not play a decisive role in the optical and photocatalytic activity of MoSe_2_. In addition, the diffraction peaks of as-prepared MoSe_2_ samples shift toward smaller angle, suggesting their larger interlayer spacing than that of the MoSe_2_ bulk.

**Figure 2:**
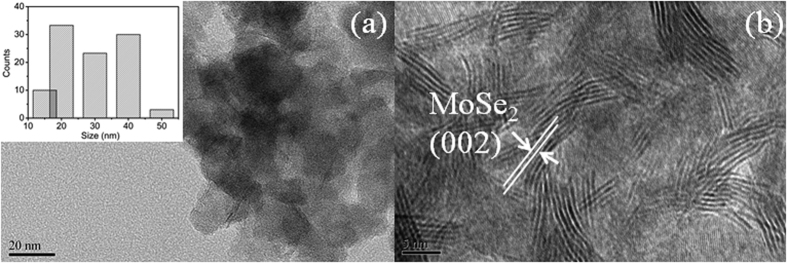
(**a**) Low-magnification and (**d**) high-magnification HRTEM images of M-2. Scale bars: (**a**) 20 nm and (**b**) 5 nm. Inset is the particle size distribution of (**a**) M-2.

**Figure 3:**
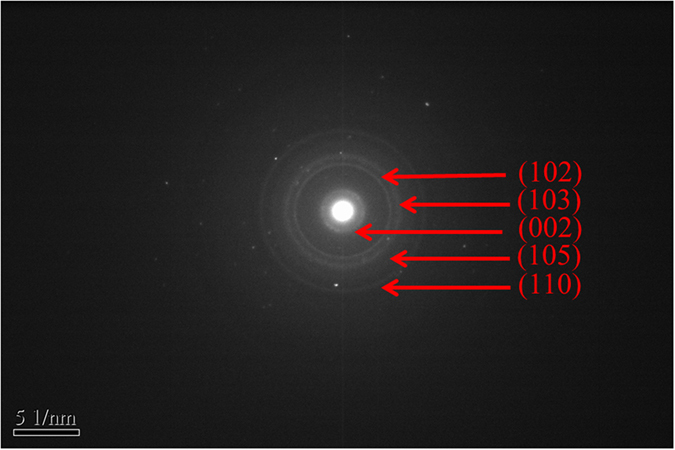
SAED pattern of M-2. Scale bars: 5 1/nm.

Figure 2a,b show the high-resolution transmission electron microscopy (HRTEM) images of M-2. The morphologies of M-1, M-3 and M-4 (not shown here) are similar to that of M-2. MoSe_2_ nanoparticles are monodisperse with the diameters in the range of 30–50 nm (inset of Fig. 2). Furthermore, it is clearly observed from Fig. 2b that the MoSe_2_ nanoparticles are formed by self-assembly nanosheets. Commonly, the MoSe_2_ appears to have a 2D sheet-like structure with abundant active defective-edges, but the defects can act as recombination center instead of providing an electron pathway and promote the recombination of electron-hole pairs^40^. Compared with MoSe_2_ with 2D sheet-like structure, the defects of MoSe_2_ nanoparticles formed by self-assembly nanosheets are relatively less, which is beneficial to the photocatalytic activity. The interlayer spacing of 0.68 nm observed from lattice fringes can be ascribed to the (002) direction of hexagonal MoSe_2_, which is slightly larger than that of the MoSe_2_ bulk (0.64 nm). This result is in agreement with the XRD result. The selected area electron diffraction (SAED) pattern in Fig. 3 shows clear diffraction rings and can be well indexed as a pure hexagonal MoSe_2_ phase, indicating a high crystallinity of MoSe_2_.

**Figure 4:**
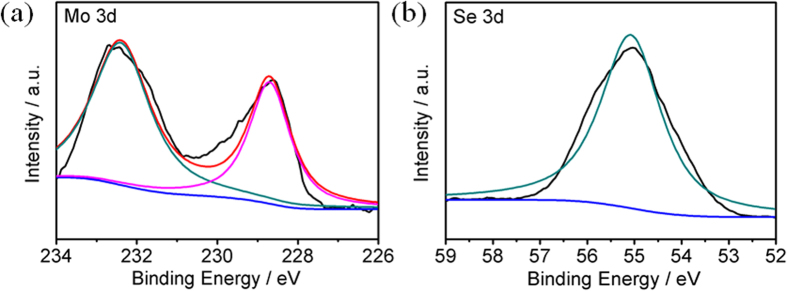
High-resolution XPS spectra of (**a**) Mo 3d and (**b**) Se 3d for M-2.

In order to investigate the chemical composition of M-2, X-ray photoelectron spectroscopy (XPS) measurements were carried out. Figure 4a, b show the high resolution XPS spectra of Mo 3d and Se 3d for M-2. Mo 3d_5/2_ and Mo 3d_3/2_ were found at 228.7 eV and 232.4 eV, respectively, revealing the chemical oxidation state of +4 for Mo and the formation of MoSe_2_^41^. The peak at 55 eV is attributed to Se 3d_3/2_. The mole ratio of Mo:Se in M-2 is about 1:2, further indicating the high purity of the products, which is in accordance with the EDS measurement. The XPS spectra of M-1, M-3, M-4 and MoSe_2_ bulk (Figure S1) are similar to that of M-2. Compared with MoSe_2_ bulk, the binding energies of Mo 3d and Se 3d for as-prepared MoSe_2_ show a blue shift, which may be due to atomic undercoordination induced local quantum entrapment and polarization^42^. All of these results clearly confirm the formation of MoSe_2_ photocatalyst.

### Ultrahigh photocatalytic activity

**Figure 5:**
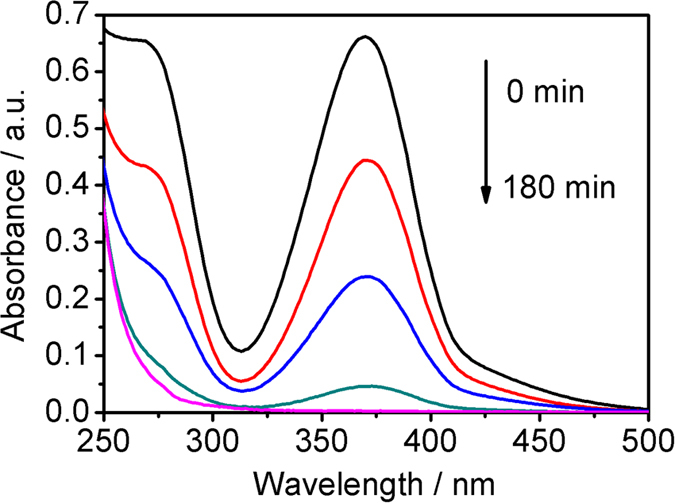
UV-vis absorbance of Cr(VI) with the variation of NIR light irradiation time.

Photocatalytic reduction of Cr(VI) by M-1, M-2, M-3 and M-4 was performed under NIR light irradiation. Figure 5 shows the UV-vis absorption spectra of Cr(VI) with irradiation time under NIR light irradiation using M-2. It is observed that the UV-vis absorption of Cr(VI), related to its concentration in the solution, becomes weak with the increase in the irradiation time.

**Figure 6:**
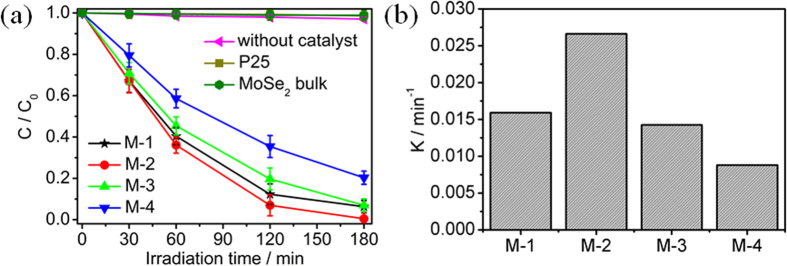
(**a)** Photocatalytic reduction of Cr(VI) by P25, MoSe_2_ bulk, M-1, M-2, M-3 and M-4 under NIR light irradiation; and (**b**) photocatalytic reaction kinetics of Cr(VI) with reaction time.

Figure 6a displays the time-dependent reduction rates of Cr(VI) by P25, MoSe_2_ bulk, M-1, M-2, M-3 and M-4 under NIR light irradiation. The normalized temporal concentration changes (*C*/*C*_0_) of Cr(VI) during the photocatalytic process are proportional to the normalized maximum absorbance (*A*/*A*_0_), which can be derived from the change in the Cr(VI) absorption profile during the photocatalysis process. It is observed that the concentration of Cr(VI) is hardly reduced under NIR light irradiation in the absence of the photocatalyst. The reduction rates of Cr(VI) for P25 and MoSe_2_ bulk are 1% and 1%, respectively. The photocatalytic activity of MoSe_2_ is dependent on the pH value of precursor solution. The reduction rate of Cr(VI) for M-1 is 94% at 180 min. When the pH value of precursor solution increases to 2, the reduction rate increases and reaches a maximum value of 100% for M-2 at 180 min. However, when the pH value of precursor solution further increases, the reduction rate decreases to 93% and 80% for M-3 and M-4 at 180 min, respectively. Figure 6b shows the linear fitting between pseudo-first-order kinetic equation and experimental data for P25, MoSe_2_ bulk, M-1, M-2, M-3 and M-4. The values of rate constants (*k*) can be obtained directly from the fitted straight-line plots of ln(*C*/*C*_0_) versus reaction time. It is observed that the values of *k* are very low (0.0002 min^−1^ for the absence of the photocatalyst; 0.0007 min^−1^ for P25 and MoSe_2_ bulk) under NIR light irradiation). The value of *k* under NIR light irradiation follows the order: M-2 (0.0267 min^−1^) > M-1 (0.0159 min^−1^) > M-3 (0.0143 min^−1^) > M-4 (0.009 min^−1^). The result shows that M-2 exhibits a best photocatalytic activity under NIR light irradiation.

**Figure 7:**
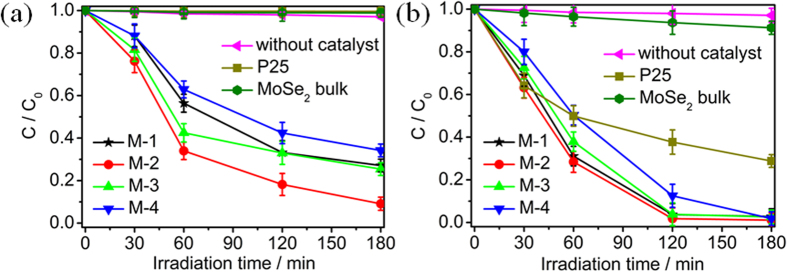
Photocatalytic reduction of Cr(VI) by P25, MoSe_2_ bulk, M-1, M-2, M-3 and M-4 under (**a**) visible and (**b**) UV light irradiation.

Photocatalytic reduction of Cr(VI) by P25, MoSe_2_ bulk, M-1, M-2, M-3 and M-4 was also performed under visible and UV light irradiation, as shown in Fig. 7. Under visible light irradiation, the reduction rates of Cr(VI) for P25, MoSe_2_ bulk, and M-1 are 1%, 1% and 73%, respectively, and a maximum reduction rate reaches 91% for M-2 at 180 min. However, when the pH value of precursor solution further increases, the reduction rate decreases to 75% and 66% for M-3 and M-4 at 180 min, respectively. Under UV light irradiation, the reduction rates are 71%, 9%, 97%, 99%, 97% and 97% for P25, MoSe_2_ bulk, M-1, M-2, M-3 and M-4, respectively. Therefore, MoSe_2_ can exhibit excellent photocatalytic activity not only under NIR light irradiation but also under UV and visible light irradiation.

**Figure 8:**
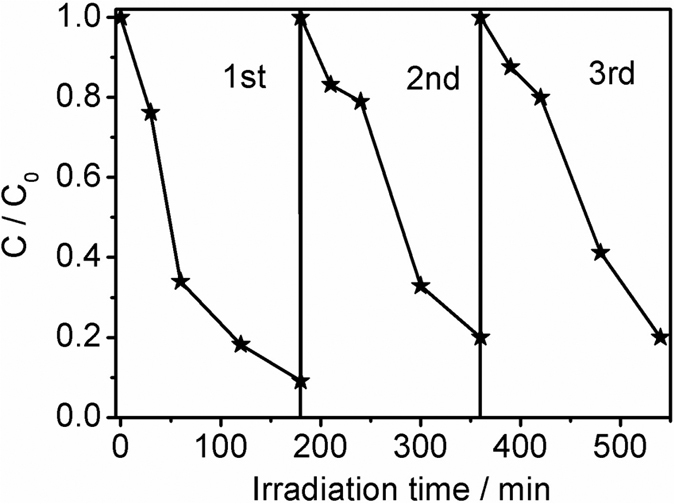
Photo-stability of M-2 by investigating its photocatalytic activity with three times of cycling uses.

The photo-stability of photocatalysts is very important for practical application. Therefore, the photo-stability of MoSe_2_ (M-2) was studied by investigating its photocatalytic activity under visible light irradiation with three times of cycling uses, as shown in Fig. 8. It is noteworthy that only insignificant decrease for photocatalytic activity is found, which may be due to the loss of photocatalyst during collection process. Moreover, the crystal structure of M-2 after the photocatalytic reaction was characterized by XRD and XPS (Figures S3 and S4). It can be observed from the XRD pattern that the crystal structure of M-2 does not show an obvious change before and after the photocatalytic reaction. The XPS spectrum of Mo 3d for M-2 after the photocatalytic reaction displays two peaks at 228.7 eV and 232.4 eV, assigned to Mo 3d_5/2_ and Mo 3d_3/2_, respectively. A characteristic peak located at 55 eV can be observed in Figure S2b, corresponding to Se 3d_3/2_. All of these results clearly confirm the good photo-stability of MoSe_2_ photocatalyst under the studied conditions.

### Mechanism of Photocatalytic Activity

**Figure 9:**
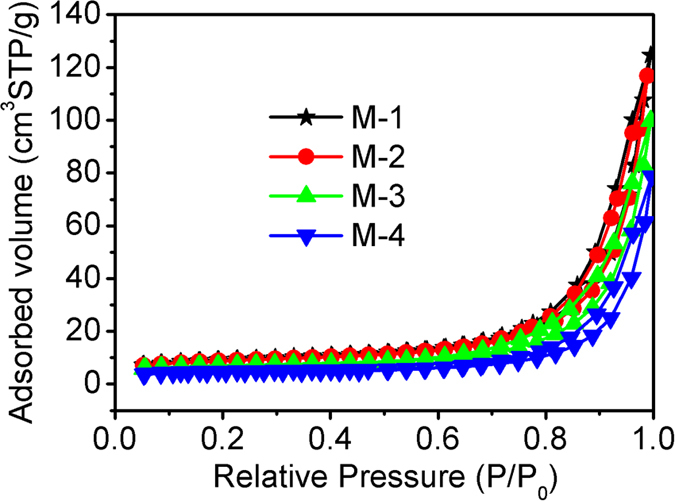
Nitrogen adsorption-desorption isotherms of M-1, M-2, M-3 and M-4.

During photocatalysis, the adsorption of Cr(VI), light absorption as well as the charge transportation and separation are crucial factors^43^,^44^. Figure 9 displays the nitrogen adsorption-desorption isotherms of M-1, M-2, M-3 and M-4. It can be observed that all of them show type IV isotherms with H3 hysteresis loop. The specific surface areas, pore sizes and pore volumes of M-1, M-2, M-3 and M-4 are listed in Table S1. The result shows that the specific surface area is almost same at low pH values of 1 and 2, while it decreases obviously when the pH value increases to 3 and 4, which may be due to the aggregation of MoSe_2_ nanoparticles. The pore volumes of M-1 and M-2 are larger than those of M-3 and M-4. Larger specific surface area and pore volume can allow more Cr(VI) to enter into the MoSe_2_, which is beneficial to the photocatalytic activity^45^. The pore size of MoSe_2_ increases with the pH value of precursor solution increases, which can influence the fast transport of Cr(VI), but it does not play a decisive role in the photocatalytic activity of MoSe_2_.

**Figure 10:**
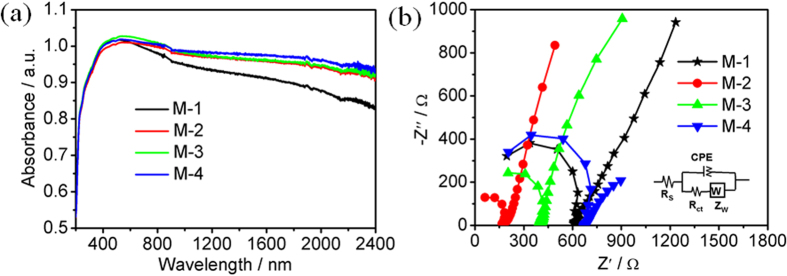
(**a**) UV-Vis-NIR diffuse absorption spectra and (**b**) Nyquist plots of M-1, M-2, M-3 and M-4. Inset of b is the corresponding equivalent circuit model.

Figure 10a shows the UV-Vis-NIR diffuses absorption spectra of M-1, M-2, M-3 and M-4. It can be observed that all MoSe_2_ samples exhibit strong absorption in the entire visible light and even NIR light region. Compared with M-1, M-2, M-3 and M-4 have better absorption. Highly ordered mesoporous crystalline MoSe_2_ synthesized using mesoporous silica SBA-15 as a hard template via a nanocasting strategy was found to show a strong absorption band covering from 400–800 nm^35^, suggesting that the highly ordered mesoporous crystalline MoSe_2_ can only absorb the visible light. Furthermore, the diffuse reflectance spectra were also used to estimate the band gap energy through the Kubelka-Munk function: F(R) = α = (1-R)^2^/2R, where R is the percentage of reflected light and α is absorption coefficient, as shown in Figure S5a. The indirect band gap of all MoSe_2_ samples are determined by extrapolating the linear portion of (αhv)^1/2^ plot (Figure S5b). The absorption edges and band gap energies of all MoSe_2_ samples are about 984 nm and 1.26 eV, respectively.

The charge transfer and recombination behavior of the as-prepared samples was studied by analyzing the electrochemical impedance spectra (EIS) spectra in dark condition. Figure 8b shows the typical Nyquist plots of M-1, M-2, M-3 and M-4. The semicircle in the EIS spectra is ascribed to the contribution from the charge transfer resistance (*R*_ct_) and constant phase element (CPE) at the photocatalyst/electrolyte interface. The inclined line, resulting from the Warburg impedance Z_W_, corresponds to the ion-diffusion process in the electrolyte. The corresponding equivalent circuit is shown in the inset of Fig. 10b. The fitted *R*_s_, *R*_ct_, CPE and Z_W_ for all MoSe_2_ samples are listed in Table S2. It is found that the *R*_ct_ for M-2 is 143.3 Ω, much lower than those for other MoSe_2_ samples, indicating that the recombination of photo-induced electrons and holes in M-2 is more effectively inhibited. The results confirm that the pH value of the precursor solution can influence the charge transfer and recombination behavior.

**Figure 11:**
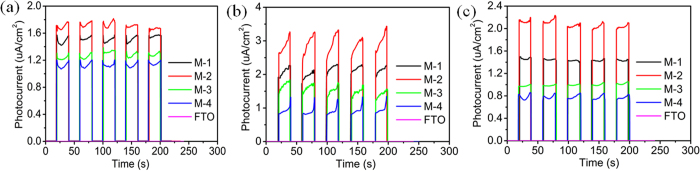
Transient photocurrent responses of M-1, M-2, M-3 and M-4 without bias versus Ag/AgCl under (**a**) UV, (**b**) visible and (**c**) NIR light irradiation.

The charge separation and transfer behavior of the as-prepared samples was also investigated by photoluminescence (PL) and photoelectronchemical measurements. Figure S6a shows PL spectra of M-1, M-2, M-3 and M-4 with the excitation wavelength of 320 nm. It can be clearly observed that the intensity of M-2 is much weaker than those of other MoSe_2_ samples, which further confirms that the recombination of photo-induced electrons and holes in M-2 can be effectively inhibited. Figure S6b shows the time resolved PL (TRPL) spectra of M-1, M-2, M-3 and M-4. The decay curves were fitted by exponentials to obtain the decay time. The average lifetimes of charge carriers are calculated to be 0.29, 0.34, 0.27 and 0.17 ns for M-1, M-2, M-3 and M-4, respectively. M-2 shows the longest lifetime, which can improve the charge separation and transfer efficiency, and thus enhance the photocurrent^22^. Figure 11 shows the transient photocurrent responses of M-1, M-2, M-3 and M-4 under UV, visible and NIR light irradiation in the photocatalytic reaction. It can be observed that the photocurrent quickly decreases to zero when the light is switched off, indicating the recombination of photo-induced electron and hole. M-2 exhibits the highest photocurrent, revealing more efficient charge transfer process and longer lifetime of the photo-induced electron-hole pairs. All results are in agreement with the EIS results.

It is known that during photocatalysis, the adsorption of pollutants, the light harvesting as well as the charge transportation and separation are crucial factors^46^. Compared with M-1, M-2, M-3 and M-4 show a higher absorption, resulting in an increase of the number of photo-generated electrons and holes. Compared with M-3 and M-4, M-2 shows larger specific surface area and pore volume, which is beneficial for adsorbing the Cr(VI). In addition, M-2 exhibits the lowest resistance and highest photocurrent, indicating that the recombination of photo-induced electrons and holes in M-2 is most effectively inhibited. All results are beneficial to the enhanced photocatalytic activity, which has been confirmed by the UV-Vis-NIR absorption, EIS and BET measurements. Therefore, among all samples, M-2 exhibits the best photocatalytic activity under UV, visible and NIR light irradiation.

**Figure 12:**
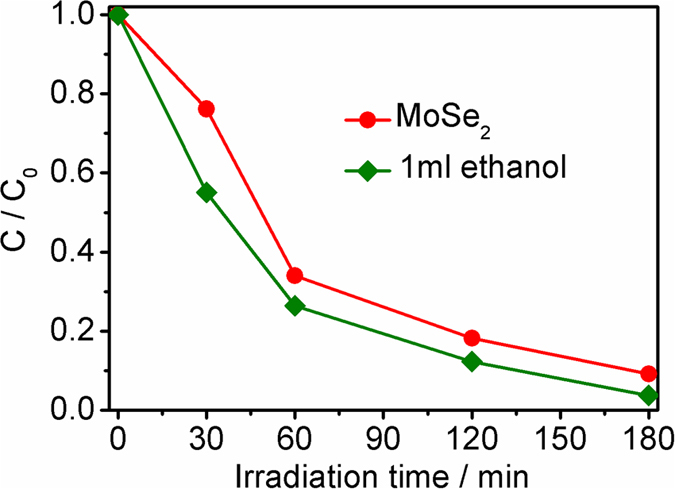
Photocatalytic reduction of Cr(VI) by M-2 with the addition of hole scavenger under visible light irradiation.

To confirm the role of the photo-generated electrons in the photocatalytic process, controlled experiments were carried out with addition of hole scavenger (ethanol). As shown in Fig. 12, the photocatalytic activity of M-2 is enhanced with the addition of hole scavenger in that the ethanol as hole scavenger can capture photo-generated holes in the photocatalytic process, and thus suppresses the recombination of photo-generated carriers. The results indicate that the photo-generated electrons govern this photocatalytic process, which is consistent with the report in the literature^47^.

**Figure 13:**
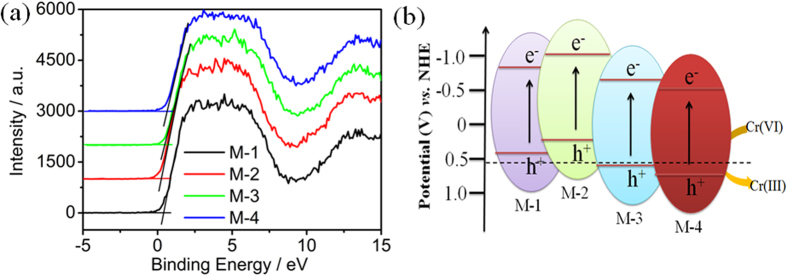
Proposed photocatalytic mechanism for MoSe_2_ under visible light irradiation.

Based on the above analysis, the possible photocatalytic mechanism of MoSe_2_ in the reduction of Cr(VI) is proposed. In the process, MoSe_2_ is excited under UV, visible or NIR light irradiation, and thus the electron-hole pairs are generated. The photo-generated charge carriers may migrate to the surface of MoSe_2_ and participate in the reduction and oxidation reactions. The valance band (VB) of all MoSe_2_ samples was measured by ultraviolet photoelectron spectroscopy (UPS) measurement, as shown in Fig. 13a. It can be observed that the VB values of M-1, M-2, M-3 and M-4 are 0.46, 0.26, 0.52, and 0.64 V (*vs*. normal hydrogen electrode (NHE)), respectively. The conduction bands (CB) are calculated to be −0.8, −1.0, −0.74, and −0.62 V (*vs*. NHE) corresponding to M-1, M-2, M-3 and M-4, respectively. The energy level structures of all MoSe_2_ samples are provided in Fig. 13b. Because the CB level of MoSe_2_ is negative than the Cr(VI)/Cr(III) potential (0.51 V, *vs*. NHE)^48^, the photo-generated electrons in MoSe_2_ can reduce the adsorbed Cr(VI) to produce Cr(III) in the photocatalytic process. Many researches indicate that the Cr(III) species will precipitate on the surface of photocatalyst as Cr_2_O_3_ or Cr(OH)_3_ in the photocatalytic process, which can be removed via simply washing with deionized water or NaOH^49^. Meanwhile, the hole can oxidize the water to form oxygen in the photocatalytic process^50^. Figure S7 shows the O_2_ evolution yield with irradiation time under visible light irradiation using M-2. It is observed that the O_2_ evolution yield increases with the increase in the irradiation time, indicating the production of O_2_ in the photocatalytic process. The major reaction steps are summarized as follows:


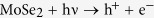



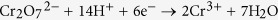



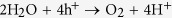


## Conclusions

MoSe_2_ samples were successfully synthesized via a facile solvothermal method and their photocatalytic activity in the reduction of Cr(VI) under UV, visible and NIR light irradiation was investigated. The results show that (i) the as-prepared MoSe_2_ exhibits excellent photo-absorption in the whole light region; (ii) MoSe_2_ samples display good photocatalytic activity with a Cr(VI) reduction rates of 99%, 91% and 98% at 180 min under UV, visible and NIR light irradiation, respectively; (iii) the enhanced photocatalytic activity is ascribed to the comparatively higher light absorption, efficient charge separation and transfer as well as the relatively large number of surface active sites; (iv) the photo-generated electrons govern this photocatalytic process.

## Methods

### Preparation of MoSe_2_

18 mmol Na_2_SO_3_, 4.5 mmol Se power, 2.25 mmol Na_2_MoO_4_ and 4.5 mmol NaBH_4_ were dissolved into 60 ml aqueous solution by sonication for 30 min to produce a uniform dispersion. A dilute HCl solution was dropped in the above solution to adjust the pH value, and the mixture was stirred for 30 min. Subsequently, the mixture was transferred into a 100 ml Teflon-lined stainless steel autoclave, and treated at 150 °C for 12 h. The MoSe_2_ samples synthesized at pH values of 1, 2, 3 and 4, named as M-1, M-2, M-3 and M-4, were isolated by filtration, washed three times with distilled water, and finally dried in a vacuum oven at 60 °C for 24 h. For the photoelectrochemical testing, 90 mg sample with 0.2 ml 2.5 wt.% polyvinyl alcohol binder was homogenously mixed in water to form slurry. Then, the resultant slurries were coated on the graphite flake (2 cm × 2 cm). Finally, these prepared electrodes were dried in a vacuum oven at 60 °C for 24 h.

### Characterization

The morphology and structure of the samples were characterized by FESEM (Hitachi S-4800), HRTEM (JEOL-2010), XRD (Holland Panalytical PRO PW3040/60) with Cu Kα radiation (V = 30 kV, I = 25 mA), and EDS (JEM-2100). XPS measurement was performed on an Imaging Photoelectron Spectrometer (Axis Ultra, Kratos Analytical Ltd.) with a monochromatic Al Kα X-ray source. The Brunauer-Emmett-Teller specific surface areas of the samples were evaluated on the basis of nitrogen adsorption isotherms measured at 77 K using a BELSORP-max nitrogen adsorption apparatus (Micrometitics, Norcross, GA). The diffuse absorption and reflection spectra of the samples were recorded using a PerkinElmer Lambda750S UV-vis-NIR spectrophotometer equipped with an integrated sphere attachment by using BaSO_4_ as a reference. PL spectra at room temperature were examined by fluorescence spectrophotometer (HORIBA Jobin Yvon fluoromax-4). The TRPL spectra were obtained on an Edinburgh Lifespec II spectrofluorometer (Edinburgh, UK). Photoelectrochemical measurements were carried out on an electrochemical workstation (AUTOLAB PGSTAT302N) using a three electrode configuration with the as-prepared films as working electrodes, a Pt foil as counter electrode and a standard calomel electrode as reference electrode. The electrolyte was 80 mg l^−1^ Cr(VI) aqueous solution. The photocurrent measurement was performed at a constant potential of +0.6 V (*vs.* SCE). 300 W Xe arc lamp (λ > 400 nm and > 800 nm) with a cut off filter and 500 W mercury lamp with a maximum emission at 356 nm were utilized as the light source. EIS were recorded in the frequency range of 0.1 Hz-1 MHz in dark conditions, and the applied bias voltage and ac amplitude were set at open-circuit voltage and 10 mV, respectively.

### Photocatalytic experiments

The photocatalytic activity of the as-prepared samples was evaluated through the experiment of photocatalytic reduction of Cr(VI) under visible and NIR light irradiation. The samples (1.2 g l^−1^) were dispersed in 80 ml Cr(VI) aqueous solutions (80 mg l^−1^) with a pH value of 7 which were prepared by dissolving K_2_Cr_2_O_7_ into deionized water. The suspensions were magnetically stirred in the dark for 30 min to reach the adsorption-desorption equilibrium. Under ambient conditions and stirring, the mixed suspensions were exposed to visible and NIR light irradiation produced by a 300 W Xe arc lamp (λ > 400 nm and > 800 nm) with a cut off filter. The light intensity of 300 W Xe arc lamp (λ > 400 nm and > 800 nm) with a cut off filter was approximately 741 and 310 mW cm^−2^ measured using a chromameter (CS-100A). A 500 W mercury lamp with a maximum emission at 356 nm and light intensity of 200 mW cm^−2^ was used as the UV source for photocatalysis. At certain time intervals, 2 ml of the mixed suspensions were extracted and centrifuged to remove the photocatalysts. The filtrates were analyzed by recording the absorption spectra of Cr(VI) using a PerkinElmer Lambda750S UV-vis-NIR spectrophotometer. The produced gas was analyzed with a gas chromatograph (GC-2014C, Shimadzu, Japan) equipped with a thermal conductivity detector, with N_2_ as the carrier gas. A recycled photocatalytic activity test was carried out according to the above-mentioned procedure. After each run of photocatalytic reaction, the fresh Cr(VI) aqueous solution was injected, and the separated photocatalyst was washed with deionized water carefully and used again. To investigate the photocatalytic mechanism, trapping experiments were carried out to determine the main reactive species in the photocatalytic process. The experimental procedure was similar to the photocatalytic activity measurement except that the hole scavengers were added into the reaction system.

## Additional Information

**How to cite this article**: Chu, H. *et al*. Hexagonal 2H-MoSe_2_ broad spectrum active photocatalyst for Cr(VI) reduction. *Sci. Rep.* **6**, 35304; doi: 10.1038/srep35304 (2016).

## Supplementary Material

Supplementary Information
