# Supplementary material for: Hexagonal 2H-MoSe2 broad spectrum active photocatalyst for Cr(VI) reduction
Source: Sci Rep. 2016 Oct 13;6:35304. doi: 10.1038/srep35304 (PMC5062113; doi:10.1038/srep35304)
Supplement: Supplementary Information [file srep35304-s1.doc]

Hexagonal 2H-MoSe_2_ broad spectrum active photocatalyst for Cr(VI) reduction

Haipeng Chu^a^, Xinjuan Liua,[[1]](#footnote-2)*, Baibai Liu^a^, Guang Zhu^b^, Wenyan Lei^a^, Huigang Du^a^, Junying Liu^d^, Jianwei Li^c^, Can Li^a^, and Changqing Sun^a^

^a^Institute of Coordination Bond Metrology and Engineering, College of Materials Science and Engineering, China Jiliang University, Hangzhou 310018, China

^b^Anhui Key Laboratory of Spin Electron and Nanomaterials, Suzhou University, Suzhou 234000, China

^c^Jiangsu Key Laboratory of Advanced Laser Materials and Devices, Hydrogen energy laboratory, Laboratory for Quantum Design of Functional Material, School of Physics and Electronic Engineering, Jiangsu Normal University, Xuzhou 221116, China

^d^Research Center for Combustion and Environment Technology, Shanghai Jiao Tong University, Shanghai 200240, China

**Supplementary Figures**

**
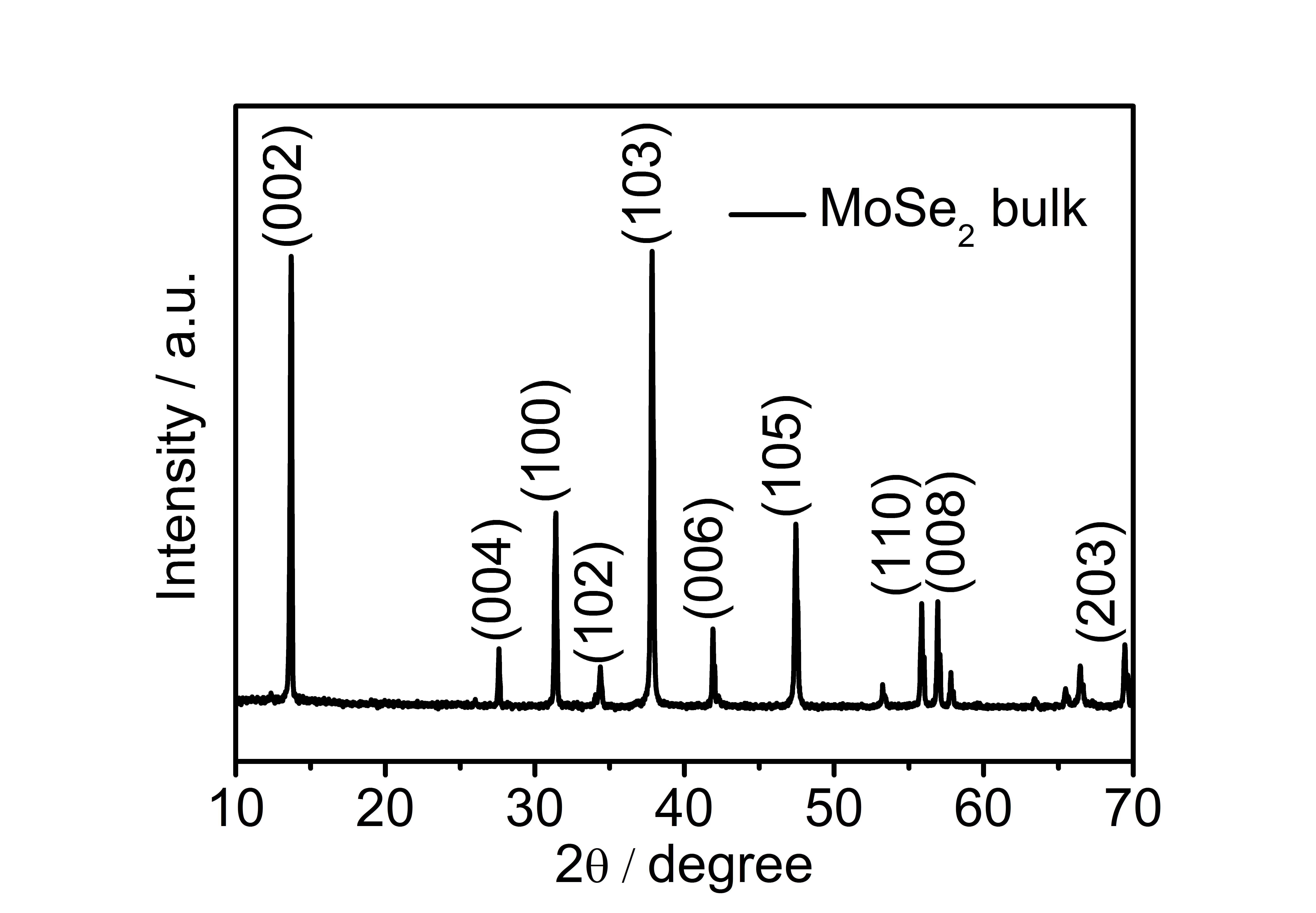
**

**Supplementary Figure 1.** XRD pattern of commercial MoSe_2_ (labeled as MoSe_2_ bulk).

**
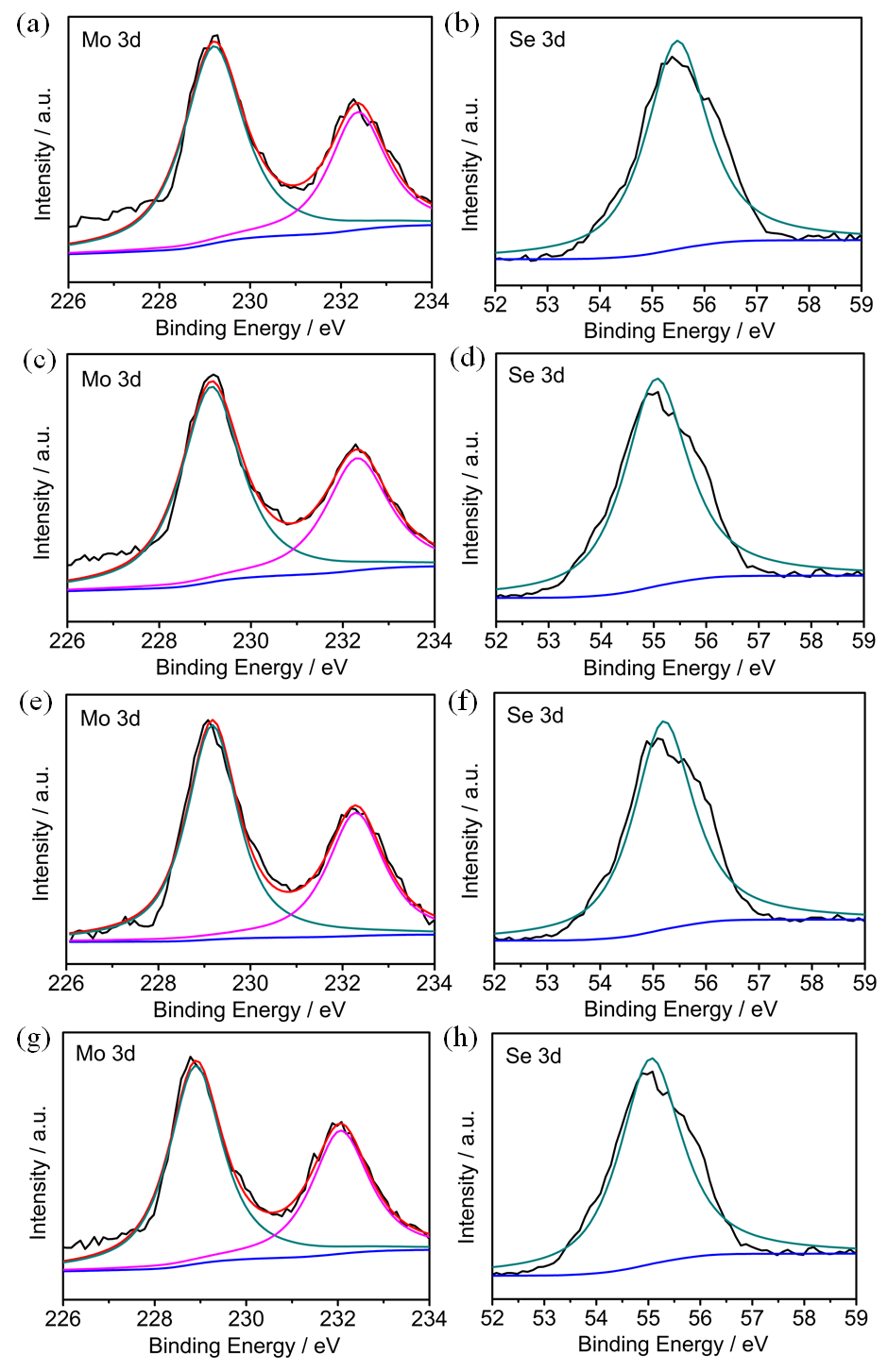
**

**Supplementary Figure 2.** High-resolution XPS spectra of Mo 3d and Se 3d for (a, b) M-1, (c, d) M-3, (e, f) M-4 and (g, f) MoSe_2_ bulk.


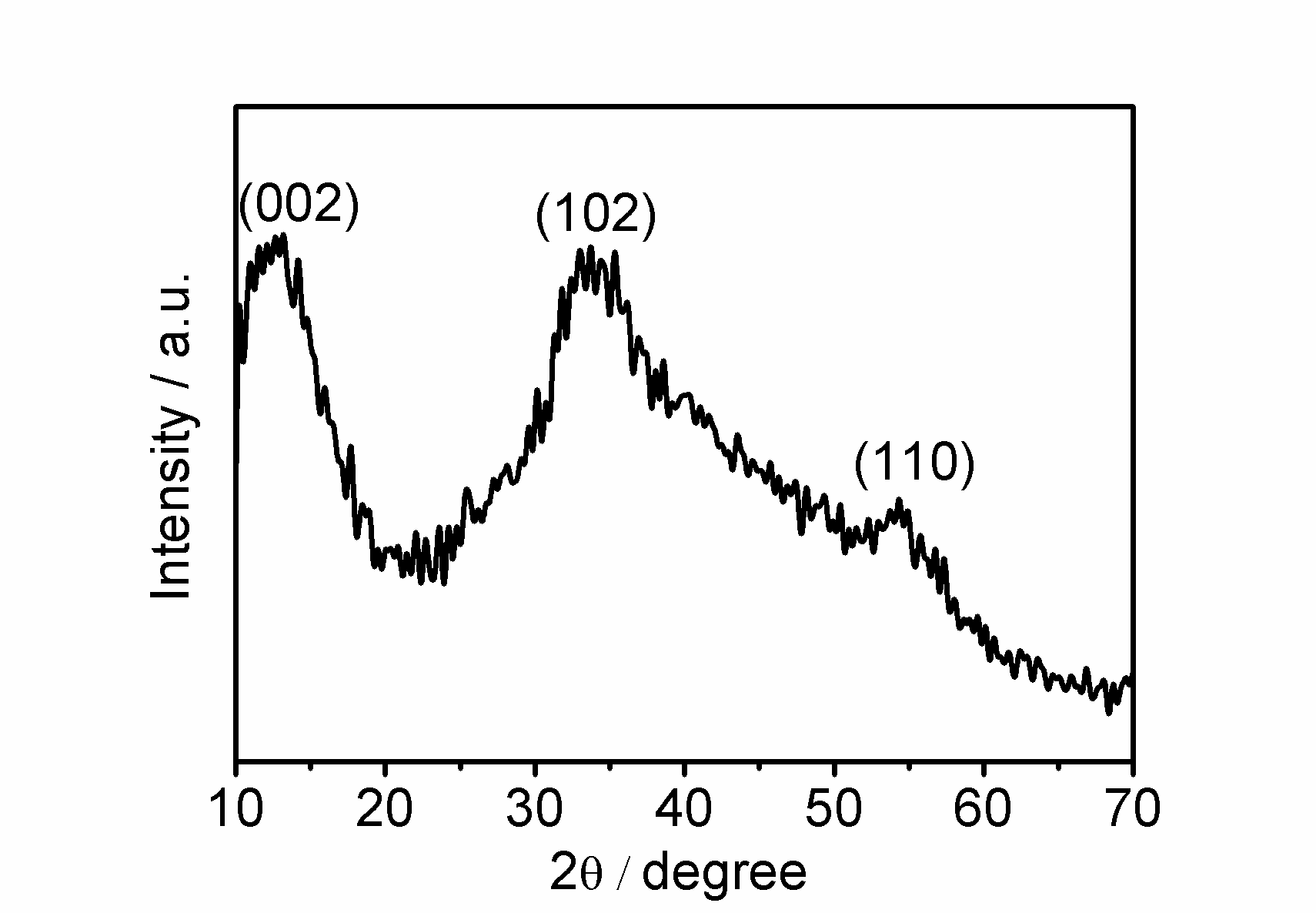


**Supplementary Figure 3.** XRD pattern of M-2 after the photocatalytic reaction.


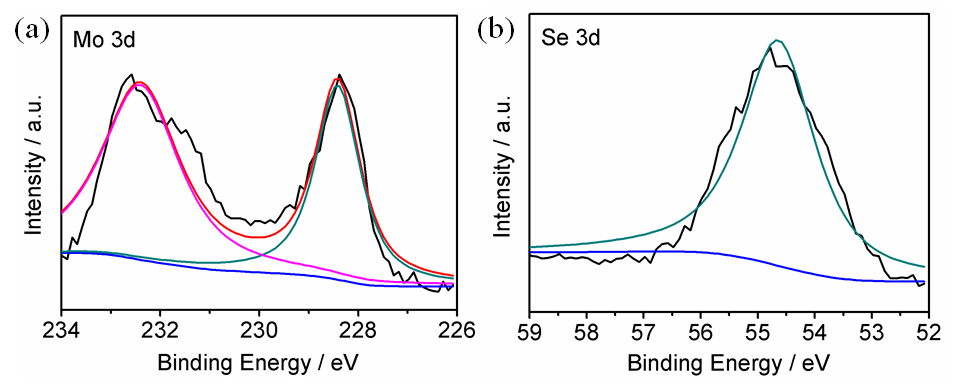


**Supplementary Figure 4.** (a-b) High-resolution XPS spectra of (a) Mo 3d and (b) Se 3d for M-2 after the photocatalytic reaction.

**
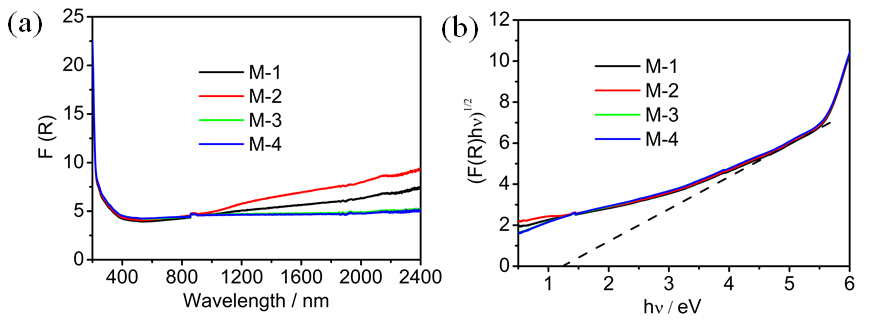
**

**Supplementary Figure 5.** (a) Diffuse reflectance spectrum and (b) Transformed Kubelka-Munk spectrum of M-1, M-2, M-3 and M-4.


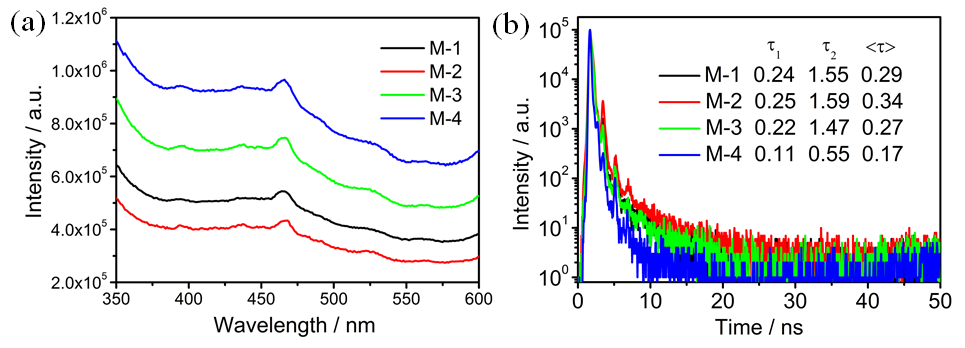


Supplementary Figure 6. (a) Steady state and (b) time resolved PL spectra of M-1, M-2, M-3 and M-4.

**
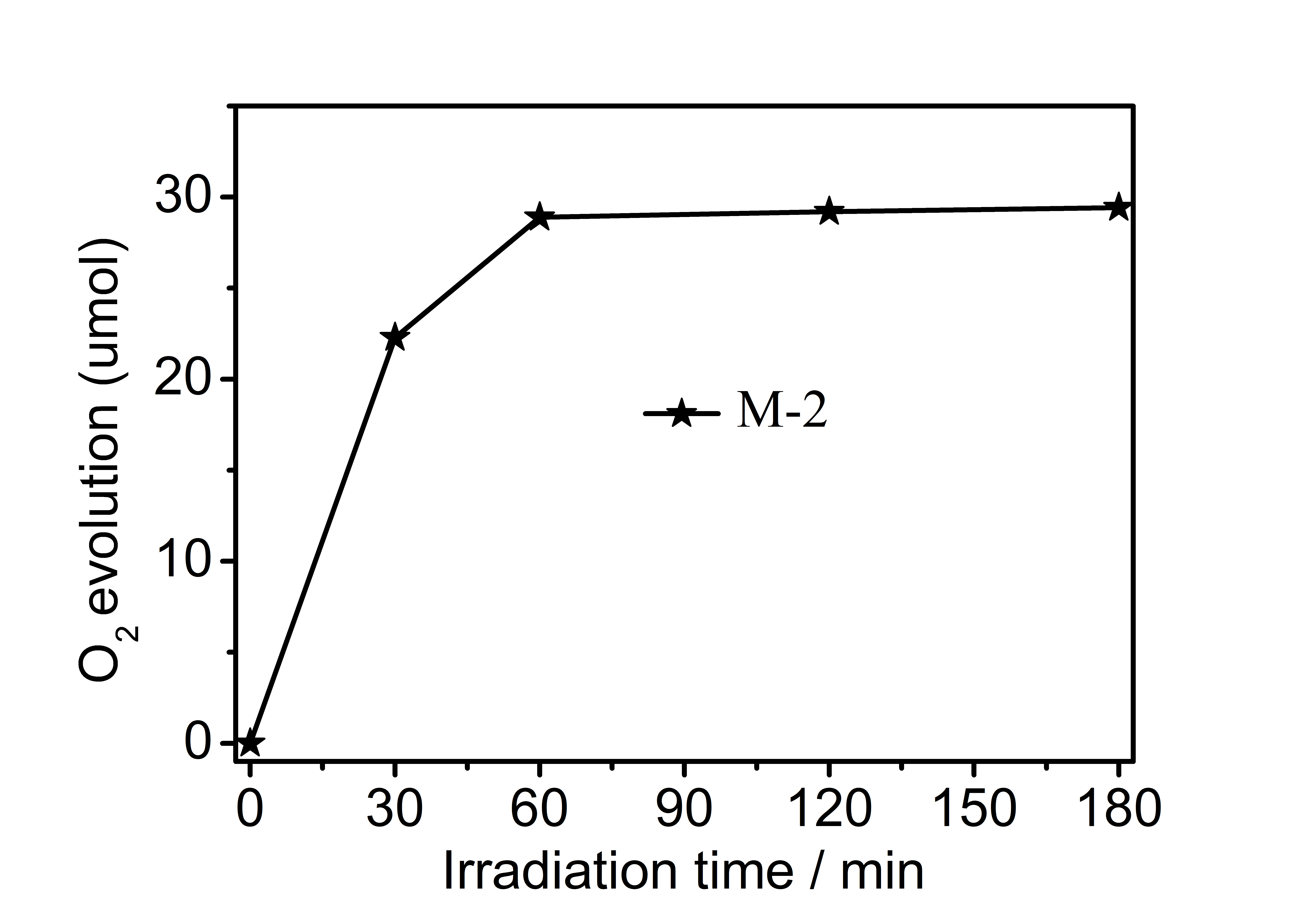
**

**Supplementary Figure 7.** O_2_ evolution yield with the variation of visible light irradiation time using M-2.

**Supplementary Table 1** Specific surface areas, pore volumes and mean pore diameters for all MoSe_2_ samples.

| Sample | M-1 | M-2 | M-3 | M-4 |
| --- | --- | --- | --- | --- |
| specific surface area (m^2^ g^-1^) | 31 | 30 | 23 | 15 |
| Pore volume (cm^3^ g^-1^) | 0.17 | 0.15 | 0.13 | 0.09 |
| Mean pore diameter (nm) | 20 | 21 | 22 | 24 |

**Supplementary Table 2** The fitted R_s_, R_ct_, and constant phase element (CPE) for all MoSe_2_ samples.

| Sample | M-1 | M-2 | M-3 | M-4 |
| --- | --- | --- | --- | --- |
| R_s_ (Ω) | 54.3 | 51.1 | 49.9 | 52.9 |
| R_ct_ (Ω) | 506.9 | 143.3 | 384.1 | 651.2 |
| CPE (F, 10^-9^) | 4.4 | 2.9 | 4.2 | 4.4 |

1. *Corresponding author. Tel: +86-571-86872475; Fax: +86-571-86872475; E-mail address: lxj669635@126.com [↑](#footnote-ref-2)
